# Simultaneous two-sided endocarditis: cardiac resynchronization leads and left atrial appendage occluder

**DOI:** 10.1007/s00392-020-01623-y

**Published:** 2020-03-05

**Authors:** Maximilian von Roeder, David Holzhey, Marcus Sandri, Holger Thiele

**Affiliations:** 1 Department of Internal Medicine/Cardiology, Heart Center Leipzig at Leipzig University, Struempellstr. 39, 04289 Leipzig, Germany; 2 Department of Cardiac Surgery, Heart Center Leipzig at Leipzig University, Leipzig, Germany

Sirs:

A 54-year-old female patient was referred to our hospital for lead extraction of a cardiac device-related infective endocarditis (CDRIE). The patient had diabetes, heart failure with reduced ejection fraction, morbid obesity (BMI 51 kg/m^2^), coronary artery disease and persistent atrial fibrillation. An originally implanted implantable cardioverter defibrillator (ICD) for primary prevention was upgraded to a cardiac resynchronization system (CRT-D) 2 years ago due to AV-node-ablation for therapy refractory atrial fibrillation. Three years ago, a left atrial appendage occluder (LAAO, Amulet, Abbott, St. Paul, MN) was implanted due to recurrent gynecological bleedings under oral anticoagulation.

**Fig. 1:**
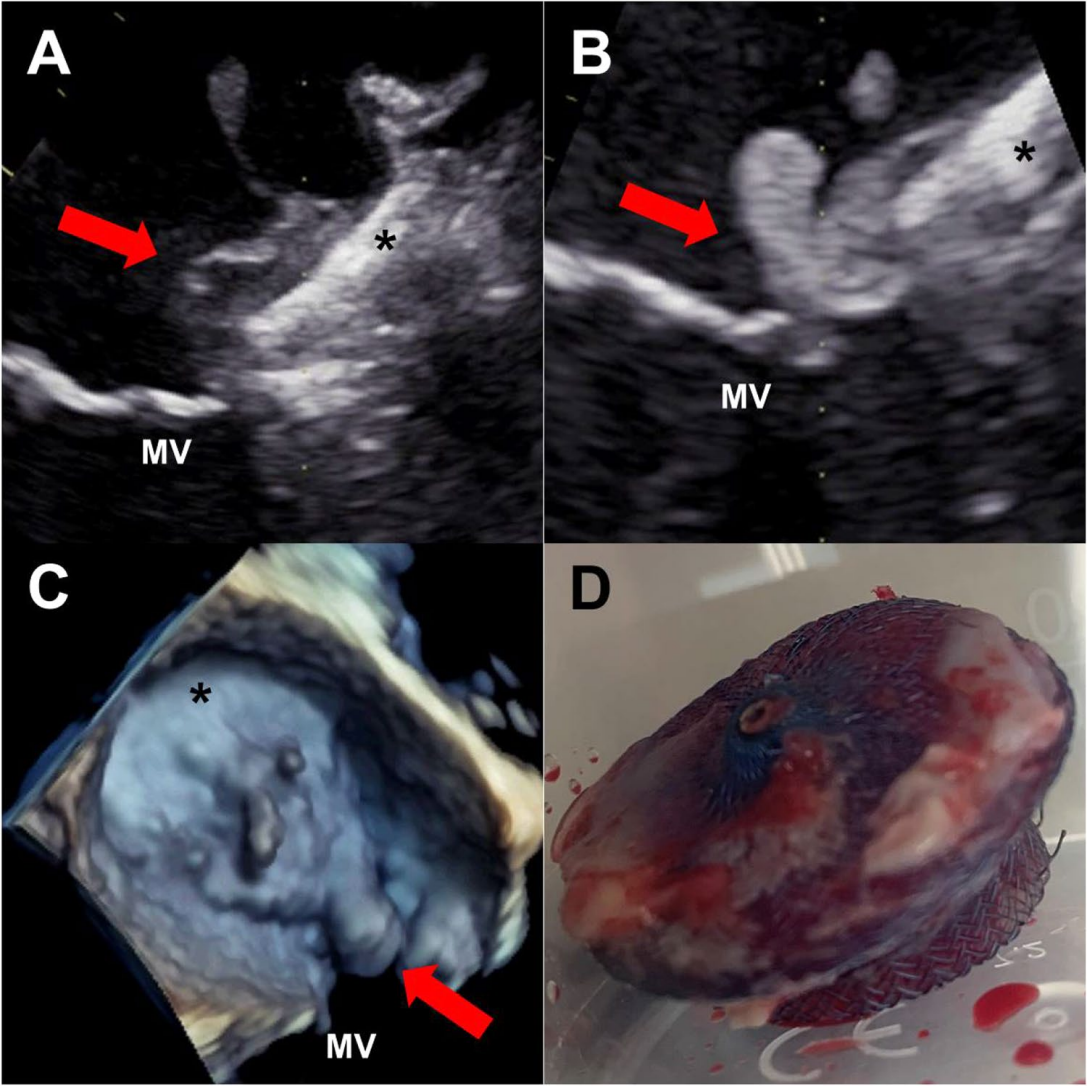
**a** Transesophageal echo (TEE) post transvenous lead extraction revealing a gelatinous mass (red arrow) on the LAAO (asterisk) in close proximity to the mitral valve (MV). **b** Control TEE showing persistence of the mass with growth (red arrow) towards the mitral valve (MV). **c** 3-D-image of the LAAO (asterisk) with the growing mass on the device and in the anterolateral commissure (red arrow) of the MV. **d** Retrieved device showing endothelial growth and purulent infection

She was admitted to the referring hospital with fever and chills. Blood cultures showed persistent bacteremia with methicillin-resistant *Staphylococcus aureus.* Transthoracic echocardiography revealed a suspicious mass on the implanted CRT lead. On admission we performed a transesophageal echocardiography (TEE) which confirmed a lead vegetation of 13 × 5 mm size but the exam was stopped prematurely due to respiratory insufficiency and given the presumed identification of the causative focus. Treatment with vancomycin was continued and transvenous lead extraction was performed successfully. Cultures of the pacemaker lead also confirmed methicillin-resistant *Staphylococcus aureus.* A second TEE was performed following complete device extraction and respiratory stabilization: no signs of valvular vegetations could be found but a gelatinous mass with floating parts (Fig. 1, panel a) was present on the LAAO. The patient was started on intravenous heparin and after 7 days TEE showed no reduction of the mass on the LAAO. Instead the vegetation increased towards the mitral valve (Fig. 1, panel b, c). Given the uncontrolled infection the patient was planned for surgery. The infected LAAO (panel d) was retrieved, the mitral valve replaced and an epimyocardial pacemaker lead was implanted on the left ventricle. Histopathology confirmed highly active purulent infection of the LAAO and the surrounding tissue. Postoperatively the patient remained hemodynamically stable and was discharged to a rehabilitation clinic with ongoing antibiotic therapy.

With the TEE confirming lead vegetation and persistent bacteremia with endocarditis-typical specimens the diagnosis endocarditis is made according to Duke criteria [1]. Even though the TEE was terminated prematurely it revealed no signs of valvular endocarditis or right sided abscess formation. Accordingly, transvenous lead extraction in addition to antibiotics is the preferred therapy in patients with CDRIE while a surgical approach remains to be considered in cases of large vegetations [2, 3]. The endocarditic involvement of the LAAO at that point of time remains unclear. To the best of our knowledge no case of an infected LAAO has been reported so far and presumed incidence must be very low. Thrombus formation on an LAAO on the other hand occurs in about 3.7% of cases [4] and antithrombotic treatment was started accordingly. However, the mass did not decrease and the patient was planned for surgery given the uncontrolled infection and the progressive involvement of the mitral valve. Whether earlier surgery would have been possible sparing the mitral valve remains open to speculation but this case should help to create awareness of this rare situation. Especially in the case of an aggressive specimen such as *Staphylococcus aureus* and given the proximity of the LAA to the mitral valve, a thorough echocardiographic exam should be mandatory.
